# A systematic review and meta-analysis of implicit Theory of Mind in schizophrenia

**DOI:** 10.1192/j.eurpsy.2022.319

**Published:** 2022-09-01

**Authors:** T. Csulak, A. Hajnal, S. Kiss, F. Dembrovszky, Z. Sipos, M. Varjú-Solymár, M. Kovács, M. Herold, E. Varga, P. Hegyi, T. Tényi, R. Herold

**Affiliations:** 1 University of Pécs, Department Of Psychiatry And Psychotherapy, Pécs, Hungary; 2 University of Pécs, Institute For Translational Medicine, Pécs, Hungary; 3 University of Pécs, Department Of Paediatrics, Pécs, Hungary

**Keywords:** Theory of Mind, mentalizing, schizophrénia, implicit

## Abstract

**Introduction:**

Everyday social interactions are based on Theory of Mind (ToM) or mentalizing, whose complex processes are involved in understanding, representing one’s own and other people’s mental states. ToM is supposed to have two systems. The implicit ToM seems to be a fast, automatic, non-verbal processing. The explicit ToM is characterized by a slower, but more flexible processing, which is mostly verbal, interpretative. Several studies have described explicit ToM deficit in schizophrenic patients. Less research has investigated implicit ToM in patients, however recently, there has been a growing number of articles examining implicit ToM of patients with schizophrenia.

**Objectives:**

The aim of our systematic review and meta-analysis is to summarize the results of the implicit ToM in schizophrenia.

**Methods:**

A systematic search was performed in four major databases. We included 11 publications. 7 studies; and 5 studies were included the quantitative synthesis and the qualitative synthesis, respectively.

**Results:**

We found significant differences in accuracy, reaction time and brain activation patterns during implicit ToM between schizophrenic patients and controls. The systematic review revealed further alterations in visual scanning, cue fixation, face looking time, and difficulties in perspective taking.

**Conclusions:**

Based on our results implicit ToM is affected in schizophrenia in addition to explicit ToM deficit. However, based on these results we cannot exclude the possibility, that implicit ToM or at least some elements of it might be relatively unaffected (e.g. detection of intentionality), however its effectiveness is limited by non-mentalizing deficits (e.g. certain neurocognitive impairments). Our results may have important implications for the remediation of mentalizing skills.

**Disclosure:**

The research is supported by the Hungarian National Excellence Centrum Grant (FIKP II) and Hungarian Brain Research Program (KTIA-13-NAP-A-II/12).

